# The Effect of Pedaling at Different Cadence on Attentional Resources

**DOI:** 10.3389/fnhum.2022.819232

**Published:** 2022-02-25

**Authors:** Mayu Akaiwa, Koki Iwata, Hidekazu Saito, Eriko Shibata, Takeshi Sasaki, Kazuhiro Sugawara

**Affiliations:** ^1^Graduate School of Health Sciences, Sapporo Medical University, Sapporo, Japan; ^2^Department of Rehabilitation, Kashiwaba Neurosurgical Hospital, Sapporo, Japan; ^3^Department of Occupational Therapy, School of Health Science, Sapporo Medical University, Sapporo, Japan; ^4^Department of Physical Therapy, Faculty of Human Science, Hokkaido Bunkyo University, Eniwa, Japan; ^5^Department of Physical Therapy, School of Health Science, Sapporo Medical University, Sapporo, Japan

**Keywords:** electroencephalography, attention, P300, pedaling, oddball paradigm

## Abstract

We investigated the relationship between attentional resources and pedaling cadence using electroencephalography (EEG) to measure P300 amplitudes and latencies. Twenty-five healthy volunteers performed the oddball task while pedaling on a stationary bike or relaxing (i.e., no pedaling). We set them four conditions, namely, (1) performing only the oddball task (i.e., control), (2) performing the oddball task while pedaling at optimal cadence (i.e., optimal), (3) performing the oddball task while pedaling faster than optimal cadence (i.e., fast), and (4) performing the oddball task while pedaling slower than optimal cadence (i.e., slow). P300 amplitudes at Cz and Pz electrodes under optimal, fast, and slow conditions were significantly lower than those under control conditions. P300 amplitudes at Pz under fast and slow conditions were significantly lower than those under the optimal condition. No significant changes in P300 latency at any electrode were observed under any condition. Our findings revealed that pedaling at non-optimal cadence results in less attention being paid to external stimuli compared with pedaling at optimal cadence.

## Introduction

Humans experience situations that require the execution of multiple tasks simultaneously. Dual tasks, where individuals perform two tasks simultaneously, degrade the performance (Al-Yahya et al., 2011; Tomporowski and Audiffren, 2014). Especially, simultaneous performance of a cognitive and a motor task is relevant in daily life. A good example includes using a smartphone while walking or driving. They increase the risk of falls and traffic accidents (Strayer and Johnston, 2001). Performance degradation was suggested to be linked to attention (Al-Yahya et al., 2011). “Attention” refers to a variety of hypothetical constructs by which the nervous system apprehends and organizes the sensory input and generates coordinated behavior (James, 2007). Attentional resources are defined as the amount of attention available to perform motor or cognitive tasks. When humans simultaneously perform two or more tasks, resources will be shared among tasks. Attentional resources, however, are limited in capacity, and simultaneous performance of two tasks causes a competition for attentional resources (Leone et al., 2017). Therefore, simultaneous performance of a cognitive and a motor task results in deterioration of performance in one or both tasks.

In previous studies, participants were imposed a dual task, namely, a cognitive task and an alternate leg movement. Performing cognitive tasks during walking reduced not only gait parameters but also the cognitive ability (Al-Yahya et al., 2011). Notably, a study suggested that gait in old healthy adults was affected more by concurrent cognitive tasks compared with that in young adults (Tomporowski and Audiffren, 2014). Lajoie et al. (2016) examined attentional requirements of walking at various speeds and reported that slow walking demonstrated a significantly longer reaction time (RT) than preferred and fast walking speeds; walking at a preferred pace also led to longer RTs than did walking at a fast pace. These results indicate that slow walking speeds require more attentional resources than fast walking. However, this relationship between the gait speed and attentional resources is debatable because the previous studies reported the behavioral change but not neurophysiological assessments. Therefore, we investigated whether attentional resources are related to cadence of a locomotor task using electroencephalography (EEG).

In our study, to clarify attentional resources during alternating movement of lower limbs as a function of gait speed, participants were asked to perform oddball tasks while pedaling on a stationary bike. Pedaling has three advantages. First, the trunk and head of the participants are stabilized during pedaling compared to walking. Second, the pedaling device is light and portable. Third, studies investigating characteristics of brain activity reported that EEG can be performed during pedaling (Bailey et al., 2008; Jain et al., 2013; Bullock et al., 2015). Therefore, we used pedaling in this study.

P300, an event-related potential (ERP) component, is suited well to testing attention. Studies have reported that the P300 peak amplitude is proportional to attentional resources devoted to a given task, and latency reflects stimulus evaluation time (Sutton et al., 1965; Kutas et al., 1977; Kok, 2001). P300 can be observed most clearly in an “oddball” task (Kok, 2001). In the oddball task, participants were presented two categories of stimuli in a random sequence, namely, a rare (i.e., target stimulus) and a frequent (i.e., standard stimulus) stimulus.

Sensory inputs, including visual, auditory, and somatosensory ones, allow detecting the changes in the environment. To effectively function in our environment, we employed a set of neural mechanisms that extract the sensory inputs, which are most relevant to our current goals (Gomez-Ramirez et al., 2016). Therefore, for the execution of appropriate behaviors, it is important to elucidate the mechanisms by which we select sensory information from each modality. Additionally, previous studies have focused on studying the attentional allocation during locomotor tasks using the visual or auditory oddball paradigm (Bullock et al., 2015; Lajoie et al., 2016; Richer et al., 2018; Bradford et al., 2019). Nevertheless, the involvement of somatosensory stimuli remained undetermined. Hence, we assessed the relationship between attentional resources and pedaling cadence by measuring the P300 amplitude and latency using a tactile oddball paradigm. In contrast, previous studies reported that the P300 amplitude is affected by loading of an ergometer, and the muscle activity increases with loading of the ergometer (Hug and Dorel, 2009; Bullock et al., 2015). Therefore, we also measured electromyogram (EMG) during pedaling because there was a possibility that the P300 amplitude is modulated by the muscle activity. We hypothesized that the P300 amplitude would increase and the latency would be earlier at high cadence, whereas the amplitude would decrease and the latency would be slower at low cadence.

## Materials and Methods

### Participants

We determined the minimum sample size of the present study using the G*power software from partial η-squared. This η-squared value was determined from the previous study (Bullock et al., 2015). The effect size was set at 0.29. From this analysis, the minimum sample size was found to be 18. We recruited 25 healthy young adults (age [mean ± standard deviation]: 22.80 ± 2.08 years; 18 men and 7 women), all of whom provided written informed consent. The study conformed to the Declaration of Helsinki and the Code of Ethics of the World Medical Association and was approved by the Ethics Committee of Sapporo Medical University (No. 1-2-62).

### Stimulation

Electrical stimuli were randomly presented to the right index and fifth finger for 0.2 ms through ring electrodes attached to the first (i.e., anode) and second (i.e., cathode) interphalangeal joints. The index finger was stimulated for the target stimulus and the fifth finger for the standard stimulus. The stimulus intensity was adjusted to three times the sensory threshold of the participants (Kida et al., 2004). Stimuli were presented at a constant 1,000 ms interstimulus interval (ISI). Target and standard stimuli were randomly presented to 50% each (Nakata et al., 2004). It is because the subjects would be fatigue by the pedaling task, and, moreover, it is necessary for as much signal averaging that evokes P300 as possible.

### Experimental Procedure

All participants performed the oddball task during either pedaling on a stationary bike or relaxing (i.e., no pedaling), and EEG was recorded. Participants fixated on the crosshairs in the middle of the screen during EEG recording. They were asked to perform two tasks, namely, motor and count tasks. In the motor task, participants were instructed to pedal on a stationary bike at optimal cadence, 30% faster than optimal cadence, and 30% slower than optimal cadence. We fixed the optimal cadence that makes the participants pedal comfortably. Participants were trained to maintain a smooth consistent pedaling cadence in synchrony with a metronome before recording EEG. In the count task, participants silently counted the number of target stimuli presented to their index finger on the stationary bike. We set them four conditions, namely, (1) performing only the count task (i.e., control), (2) performing the count task during pedaling at optimal cadence (i.e., optimal), (3) performing the count task while pedaling faster than optimal cadence (i.e., fast), and (4) performing the count task during pedaling slower than optimal cadence (i.e., slow). One run varied randomly between 80 and 120 epochs (mean: 100 epochs), so the participants could not estimate the number of target stimuli. All participants performed three runs at each condition (i.e., 300 epochs in total). The order of these tasks was randomized for each run. Participants had time to recover between runs. We used the modified Borg scale to measure the perceived intensity of physical activity of the participants. We recorded the rating of perceived exertion before and after each run. Before the recording session was started, all participants practiced motor tasks until they felt comfortable.

### Recording and Analysis

Using the Neuropack system (Nihon Kohden, Tokyo, Japan), PowerLab, and LabChart software (ADInstruments, Dunedin, New Zealand), EEG and EMG signals were digitized and recorded. A study reported that P300 amplitudes are observed better on the midline, especially Pz (Simson et al., 1977). EEG was performed by using Ag/AgCl electrodes placed over three scalp sites, namely, Fz, Cz, and Pz, according to the International 10–20 system. Each scalp electrode was referenced to linked earlobes (i.e., A1A2). Electrode impedance was maintained below 5 kΩ at all recording sites. The electrooculogram (EOG) was recorded from the right suborbital region. Trials in which the EOG waveform exceeded 80 μV were rejected (Kida et al., 2004). Subsequently, we removed noisy epochs identified using a threshold of ±200 μV for other non-ocular artifacts (Bradford et al., 2019). EEG signals were recorded with bandpass filter 0.1–300 Hz at a sampling rate of 1,000 Hz and analyzed with low-pass filtering at 100 Hz. We analyzed the waveform evoked by the target and standard stimulus. The analysis period of ERPs ranged from 100 ms before to 500 ms after stimulus onset. The 100 ms period before stimulus onset was used as baseline. P300 amplitudes were measured from baseline to peak. Peak amplitudes and latencies of P300 were measured at 250–500 ms (Kida et al., 2003; Akaiwa et al., 2020).

Pedaling could lead to ocular artifacts that synchronize with movement (Savic et al., 2021). Therefore, we conducted an additional correlation analysis between the EEG channels (i.e., Fz, Cz, and Pz) and EOG (Li et al., 2009; Savic et al., 2021). The EOG channel was processed in the same method as the EEG channel; the averaging signal was segmented using the same analysis period and filtered in the same range used for ERP extraction. Pearson’s correlation coefficients were calculated between each condition (i.e., control, optimal, slow, and fast) for EEG channels and EOG.

**FIGURE 1:**
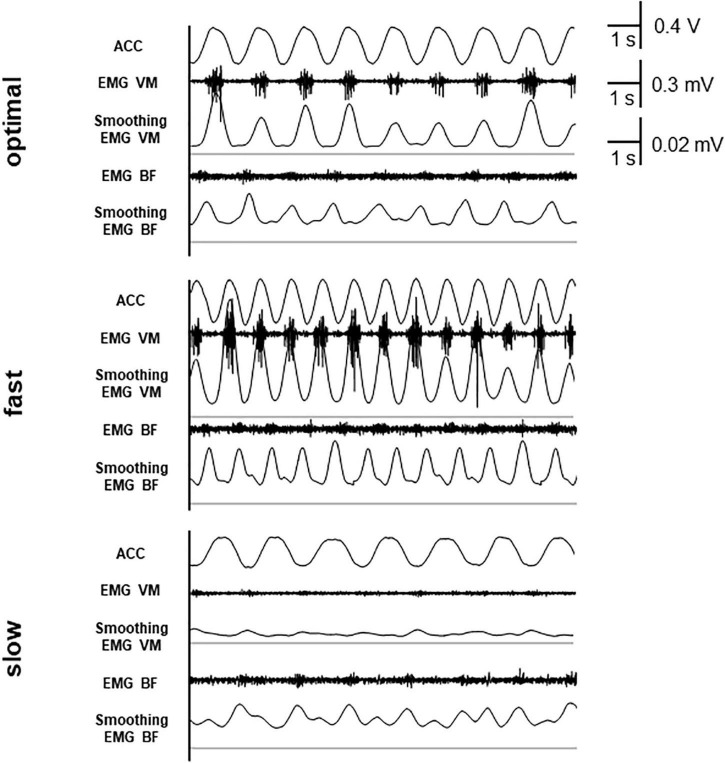
Waveforms of acceleration (ACC), rectified electromyogram (EMG), and smoothed EMG. The thin line shows the baseline (0 mV) of smoothed EMG.

Electromyogram was measured using a pair of Ag/AgCl electrodes (Blue-sensor NF-00; Ambu, Copenhagen, Denmark) mounted over the right vastus medialis (VM) and the short head of biceps femoris (BF). EMG signals (DL-140; 4 assist, Japan) were sampled at 1,000 Hz (Power Lab; AD Instruments, Dunedin, New Zealand) and bandpass filtered at 1–300 Hz. Then, the full-wave rectification and smoothing were performed. The values of moving window were 501 ms. EMG signals were normalized to maximum voluntary contractions. The averages of normalized EMG values over three pedal cycles were calculated. Figure 1 shows waveforms of acceleration, rectified EMG, and smoothed EMG at each condition in a typical participant. We assessed error rate using the following formula: Error rate = (1 – reported count/correct count) × 100.

Statistical analyses were performed using the IBM SPSS 25 software (IBM Corp., New York, NY, United States), and all data were expressed as the mean ± SD. The Shapiro–Wilk test was used to assess the normality. To analyze the assumption of sphericity prior to repeated measures ANOVA, Mauchly’s test of sphericity was used; if the result of the test was significant, indicating that the sphericity assumption was violated, the Greenhouse–Geisser adjustment was used to correct this violation. The paired *t*-test was performed on P300 under two stimuli between the target and standard. Then, repeated measures ANOVAs were performed to determine the effect of each condition (i.e., control, optimal, fast, and slow) on the amplitude and latency of P300 elicited by target and standard stimuli, the correlation coefficients between EEG and EOG, EMG, error rate, and physical activity intensity. The *post-hoc* tests were performed for significant differences in ANOVAs, with the Bonferroni correction for multiple comparisons. In addition, we analyzed bivariate correlations between P300 amplitudes and EMG activities. The significance was set at *p* < 0.05.

## Results

Optimal, fast, and slow cadence were 51.56 ± 9.67, 65.76 ± 13.99, and 35.88 ± 7.11 rpm, respectively. The Shapiro–Wilk test confirmed that all data, except physical activity intensity and %EMG, were normally distributed.

### P300

**FIGURE 2:**
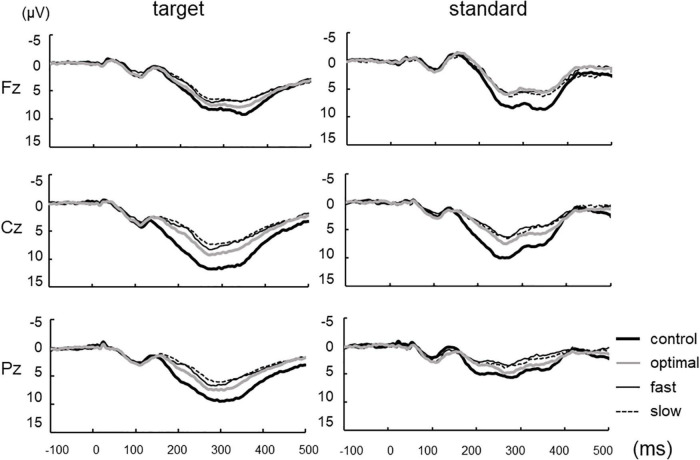
Grand-averaged waveforms of P300 elicited by target and standard stimuli at three cortical electrodes under each condition.

**FIGURE 3:**
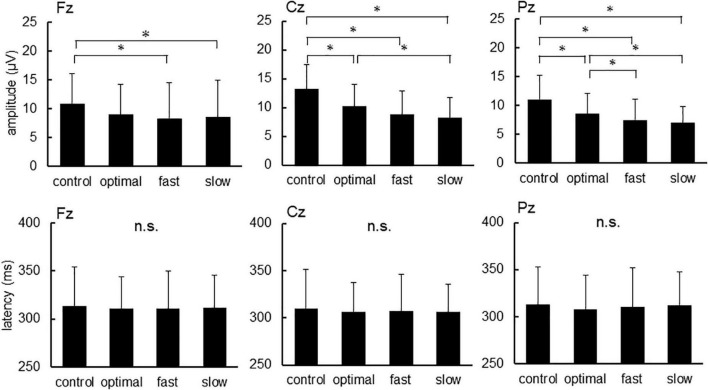
The amplitude and latency of P300 at each condition. Asterisks indicate significant differences (*p* < 0.05).

Figure 2 shows grand-averaged standard and target ERP waveforms for all participants. The average of more than 100 recordings was obtained during each condition for all participants. Repeated measures ANOVAs were used to assess the effects of cadence. There were significant main effects of each condition on P300 amplitude elicited by target stimuli [Fz, *F*_(3,22)_ = 4.047, *p* = 0.011; Cz, *F*_(3,22)_ = 21.057, *p* < 0.001; Pz, *F*_(3,22)_ = 16.551, *p* < 0.001] but not P300 latency (Figure 3; Fz, *p* = 0.900; Cz, *p* = 0.824; Pz, *p* = 0.698). *Post-hoc* tests revealed that P300 amplitude at Fz decreased under fast and slow conditions compared with that under the control condition (control, 10.92 ± 5.18 μV; fast, 8.62 ± 6.29 μV; slow, 8.32 ± 6.14 μV; control vs. fast, *p* = 0.041; control vs. slow, *p* = 0.015). P300 amplitude at Cz decreased under optimal, fast, and slow conditions compared with that under the control condition (control, 13.20 ± 4.30 μV; optimal, 10.23 ± 3.87 μV; fast, 8.82 ± 4.10 μV; and slow, 8.32 ± 4.43 μV; control vs. fast, *p* < 0.001; control vs. optimal, *p* < 0.001; control vs. slow, *p* < 0.001) and under the slow condition compared with that under the optimal condition (*p* = 0.037). P300 amplitude at Pz decreased under optimal, fast, and slow conditions compared with that under the control condition (control, 11.04 ± 4.14 μV; optimal, 8.54 ± 3.55 μV; fast, 7.49 ± 3.57 μV; and slow, 6.96 ± 2.76 μV; control vs. fast, *p* < 0.001; control vs. optimal, *p* < 0.001; control vs. slow, *p* < 0.001) and under fast and slow conditions compared with that under the optimal condition (optimal vs. fast, *p* = 0.044; optimal vs. slow, *p* = 0.030).

The P300 amplitude of all conditions, except the control and slow conditions at Fz, decreased under standard stimuli in contrast to the target stimuli (Fz: control, *p* = 0.098; optimal, *p* = 0.029; fast, *p* = 0.041; slow, *p* = 0.128; Cz: control, *p* < 0.001; optimal, *p* < 0.001; fast, *p* = 0.007; slow, *p* = 0.006; Pz: control, *p* < 0.001; optimal, *p* < 0.001; fast, *p* < 0.001; slow, *p* < 0.001). No significant differences were found in the latencies of P300. In contrast, the different conditions exerted significant effects on P300 amplitude [Fz, *F*_(3,22)_ = 9.043, *p* < 0.001; Cz, *F*_(3,22)_ = 14.671, *p* < 0.001; Pz, *F*_(3,22)_ = 9.283, *p* < 0.001]. When evoked by standard stimuli, P300 amplitude decreased under optimal, fast, and slow conditions compared with that under the control condition (Fz: optimal, *p* < 0.002; fast, *p* < 0.001; slow, *p* = 0.001; Cz: optimal, *p* < 0.001; fast, *p* < 0.001; slow, *p* = 0.004; Pz: fast, *p* = 0.002; slow, *p* < 0.001), with no significant differences among the optimal, fast, and slow conditions. No significant differences were found regarding the latencies of P300.

We calculated the Pearson’s correlation coefficients between all EEG channels and EOG (Fz: control, 0.474 ± 0.304; optimal, 0.509 ± 0.324; fast, 0.516 ± 0.316; slow, 0.492 ± 0.301; Cz: control, 0.428 ± 0.251; optimal, 0.394 ± 0.255; fast, 0.376 ± 0.270; slow, 0.443 ± 0.258; Pz: control, 0.475 ± 0.247; optimal, 0.402 ± 0.295; fast, 0.354 ± 0.289; slow, 0.436 ± 0.248). The different conditions had no significant effects on the correlation coefficients. These findings suggest that pedaling did not lead to ocular artifacts that synchronize with movement nor did it have any effect on P300.

### Electromyogram

**FIGURE 4:**
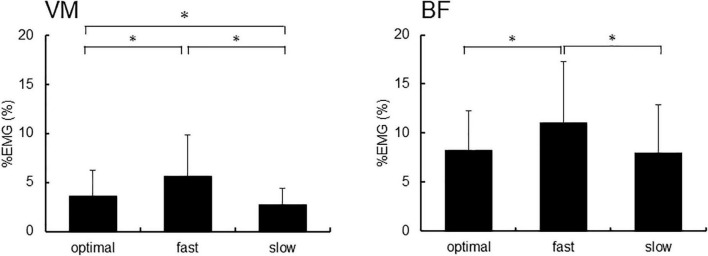
The %EMG of the right vastus medialis (VM) and the short head of biceps femoris (BF). Asterisks indicate significant differences (*p* < 0.05).

**FIGURE 5:**
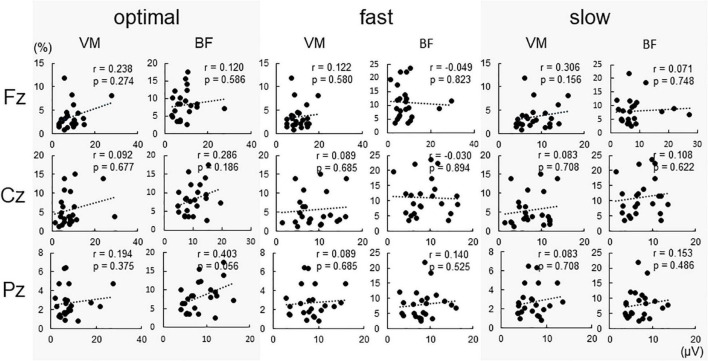
Correlations between conditions and P300 amplitude at each electrode. No significant correlations were found under all conditions.

Figure 4 shows %EMG of VM and BF. The Friedman test revealed a significant difference in %EMG of VM and BF due to conditions (VM, *p* < 0.001; BF, *p* = 0.005). The *post-hoc* analysis showed that the EMG activity in VM increased under the fast condition compared with that under slow and optimal conditions (fast vs. optimal, *p* = 0.008; fast vs. slow, *p* < 0.001) and under the optimal condition compared with that under the slow condition (optimal vs. slow, *p* = 0.018). When the relationship between P300 amplitude and %EMG was analyzed by Spearman’s rank correlation test, no significant correlation under any condition was observed (Figure 5).

### Error Rate and Intensity of Physical Activity

**FIGURE 6:**
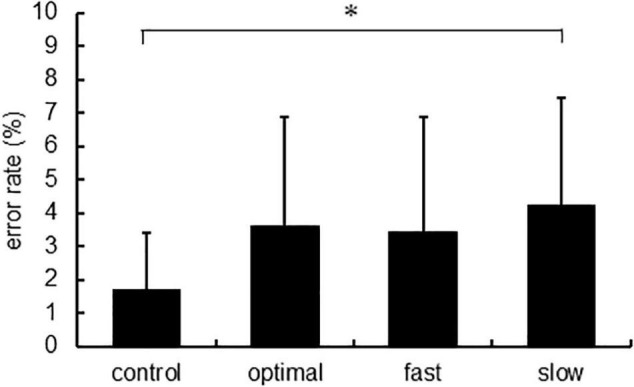
The error rate of each condition. Asterisks indicate significant differences (*p* < 0.05).

Figure 6 shows the error rate, and Table 1 shows the intensity of physical activity. There were significant main effects of each condition on the error rate [*F*_(3,22)_ = 5.020, *p* = 0.003]. The *post-hoc* analysis showed that the error rate increased under the slow condition compared with that under the control condition (*p* = 0.005). Intensities of physical activity were not significantly different under any condition.

**TABLE 1:** Rating of perceived exertion under each condition.

	Modified Borg scale	
	Median	Range
Control	0	0–0.5
Fast	0	0–2
Slow	0	0–1
Optimal	0	0–1

## Discussion

The results showed that (1) P300 amplitudes elicited by target stimuli decreased under optimal, fast, and slow conditions relative to the control condition at Cz and Pz, (2) P300 amplitudes elicited by target stimuli decreased under fast and slow conditions compared with that under the optimal condition at Pz, and (3) P300 amplitudes elicited by target stimuli were not significantly different between fast and slow conditions at Fz, Cz, and Pz. Our results showed that the optimal cadence is slow compared with that in previous studies (Ludyga et al., 2017). It could be because the participants performed pedaling on the stationary bike like a bicycle in previous studies, while performed on the reclining chair in this study.

Studies have reported that P300 amplitude is proportional to the amount of attentional resources devoted to a given task (Sutton et al., 1965; Kok, 2001) and decreases in more difficult tasks, such as dual tasks compared with a single task (Kida et al., 2012). Just et al. (2001) reported that brain activation distributes between two tasks in dual tasks. Our results suggested that attentional resources, which were shared between pedaling and counting, contributed to the reduction in the P300 amplitude.

In contrast, the P300 amplitude elicited by target stimuli at Pz under fast and slow conditions decreased compared with that under the optimal condition. A study suggested that pedaling at optimal cadence may be a good reflection of movement frequency output generated by the central pattern generator (CPG; Hansen, 2015). CPG, which is composed of spinal interneuronal networks, is a major component of the rhythm-generating system (Rossignol et al., 2006; Takakusaki, 2013). Both descending supraspinal drive and sensory feedback assist in fine-tuning the output from CPGs (Rossignol et al., 2006; Takakusaki, 2013). Moreover, studies have reported that the activation of frontal and parietal lobes increased when the count task was performed (Nieder, 2004; Šveljo et al., 2012). The sensory input during the count task may be integrated at frontal and parietal lobes through the peripheral nerve, spinal cord, thalamus, and primary somatosensory cortex. Pedaling at optimal cadence may demand a greater contribution of CPG, thus, motor control at the cerebral cortex declines. Our results showed that attentional resources for the count task decreased under fast and slow conditions possibly because motor control increased at the cerebral cortex. Therefore, pedaling at non-optimal cadence demanded greater attentional resources than at optimal cadence, and attentional resources allocated to external stimuli decreased. However, it is our limitation that the slow or fast condition could be not only a dual task, but a multitask situation, i.e., motor activity, controlling the pace, counting the targets.

In this study, P300 amplitudes elicited by target at all electrodes were not significantly different between fast and slow conditions. We hypothesized that the P300 amplitude increased under the fast condition and decreased under the slow condition (Lajoie et al., 2016). In that study, the participants performed RT tasks with auditory stimuli. Their results showed that compared with slow and self-selected speeds, accelerated walking generated faster RTs. The slowing of gait has been shown to result in greater lateral instability, increased attentional cost, reduced trunk smoothness, increased stride time variability, altered muscular activity, and higher energy costs, suggesting that slow walking increases equilibrium demands and decreases energy efficiency (Lowry et al., 2012). Thus, attentional resources during walking are decided by levels of posture control because gait speeds and posture control have a close relationship. However, in this study, participants performed alternating movement of the lower limbs on the reclining chair; therefore, the factor of posture control may be removed. For the reasons, a difference between the hypothesis and results may occur, irrespective of whether the task demands posture control.

In this study, the P300 amplitude decreased under standard stimuli compared with that under target stimuli. These results indicate that the oddball paradigm was successfully implemented. However, there were no significant differences between the control and slow conditions on Fz. The frontal component could reflect novel events or passive attention. The posterior component could reflect active attention or activation by task-relevant events, such as targets (Kok, 2001). In our count task, subjects were required to count the number of target stimuli; thus, the participants likely allocated their active attention to target stimuli rather than standard stimuli. Fz, which reflects passive attention, might not be the cause of the significant difference in the allocation of attention between target and standard stimuli compared with other EEG channels. Another finding was that the P300 amplitude evoked by standard stimuli decreased under optimal, fast, and slow conditions compared with that under the control condition. These results are similar to the P300 amplitude evoked by the target. When performing dual tasks, simultaneous tasks cause competition for attentional resources (Leone et al., 2017). Therefore, when subjects performed the count task during pedaling, the attention allocated to the counting task decreased not only for the target but also for the standard stimuli. The P300 amplitude evoked by target stimuli decreased under fast and slow conditions compared with that under the optimal condition. In contrast, there were no significant differences evoked by standard stimuli. Thus, the attention allocated to standard stimuli was not affected by the cadence of pedaling. Therefore, the P300 amplitude under optimal, fast, and slow conditions is modulated by attention allocated to the motor tasks rather than attention allocated to standard stimuli.

Our results revealed that EMG activities in the VM and BF increased under the fast condition compared with slow and optimal conditions. A study reported that the value of EMG is decided by the activation of the primary motor cortex (Cheney and Fetz, 1980), and P300 amplitudes decrease with increasing activation of the primary motor cortex (Aliakbaryhosseinabadi et al., 2017). We suggested that a decrease in P300 amplitudes under the fast condition was caused by the increased activation of the motor cortex during pedaling at high cadence.

The error rate of the slow condition was the highest; thus, slow conditions may be the most difficult tasks. A study revealed that executing smooth rhythmic motions very slowly is challenging for humans (Park et al., 2017). Similarly, it may be difficult to perform pedaling at a low cadence. This could suggest that the slow condition was a more complex task than optimal and fast conditions; thus, pedaling at low cadence results in less attention being paid to external stimuli compared with pedaling at optimal cadence.

In this study, no significant changes in the P300 latency were observed under any condition. Studies have reported that the P300 latency is related to stimulus evaluation (Kutas et al., 1977). Kida et al. (2003) showed that the P300 latency measured during ignoring the stimulus task, counting task, or reaction task is not different. Therefore, it is perhaps the P300 latency was not affected by characteristics of tasks, such as pedaling at different cadence. Our results showed that no significant correlation between P300 amplitudes and EMG activities existed under any condition. Thus, in this study, the P300 amplitude and muscle activation may be independent.

## Limitation

This study has several limitations. First, this study has not considered the effect of arousal. The P300 amplitude is associated with arousal (Polich, 2007). While, it is possible that pedaling cadence is associated with arousal, which is thought to expand the availability of attentional resources. Thus, it is possible that any relationship between pedaling cadence and P300 amplitude is due to changes in arousal. Second, our results, the P300 amplitude, would not reflect the oddball response but instead processes related to counting and memory updating. This study adopted the count task; participants counted the number of target stimuli, but this task would appear to rely on working memory (i.e., holding the current count in their mind and updating it). Third, we were unable to assess hormonal levels. In previous studies, it was shown that the female menstrual cycle can influence both attentional and motor performance (Johnston and Wang, 1991; Landen et al., 2021). Moreover, arousal is notably regulated by the progesterone and estrogen activity (Lusk et al., 2017). Therefore, differences in hormonal conditions could represent a confounding factor. Nevertheless, we verbally assessed the physical condition and arousal level of each participant before the experiment. If a participant was not feeling well or displayed a low arousal level, she/he was excluded, or the experiment was postponed.

## Conclusion

This study assessed the relationship between cadence of alternating movement of lower limbs and attentional resources. Our findings indicated that pedaling at non-optimal cadence results in less attention being paid to external stimuli compared with pedaling at optimal cadence.

## Data Availability Statement

The original contributions presented in the study are included in the article/supplementary material, further inquiries can be directed to the corresponding author.

## Ethics Statement

The studies involving human participants were reviewed and approved by the Sapporo Medical University (No. 1-2-62). The patients/participants provided their written informed consent to participate in this study.

## Author Contributions

MA, KS, and HS performed the material preparation and data collection. MA, KI, and TS performed the formal analysis. MA wrote the first draft of the manuscript. KS wrote the review and editing of the manuscript. All authors commented on previous versions of the manuscript, contributed to the study conception and design, and read and approved the final manuscript.

## Conflict of Interest

The authors declare that the research was conducted in the absence of any commercial or financial relationships that could be construed as a potential conflict of interest.

## Publisher’s Note

All claims expressed in this article are solely those of the authors and do not necessarily represent those of their affiliated organizations, or those of the publisher, the editors and the reviewers. Any product that may be evaluated in this article, or claim that may be made by its manufacturer, is not guaranteed or endorsed by the publisher.
